# Novel phthalimide based analogues: design, synthesis, biological evaluation, and molecular docking studies

**DOI:** 10.1080/14756366.2019.1637861

**Published:** 2019-07-09

**Authors:** Ismail M. M. Othman, Mohamed A. M. Gad-Elkareem, Mohamed El-Naggar, Eman S. Nossier, Abd El-Galil E. Amr

**Affiliations:** a Department of Chemistry, Faculty of Science, Al-Azhar University, Assiut, Egypt;; b Department of Chemistry, Faculty of Science and Arts of Baljurashi, Albaha University, Saudi Arabia;; c Chemistry Department, Faculty of Sciences, University of Sharjah, Sharjah, UAE;; d Pharmaceutical Medicinal Chemistry Department, Faculty of Pharmacy (Girls), Al-Azhar University, Cairo, Egypt;; e Pharmaceutical Chemistry Department, Drug Exploration & Development Chair (DEDC), College of Pharmacy, King Saud University, Riyadh, Saudi Arabia;; f Applied Organic Chemistry Department, National Research Centre, Giza, Egypt

**Keywords:** Phthalimide, antimicrobial and anticancer activities, drug-likeness, molecular modeling study, DNA gyrase B, VEGFR-2

## Abstract

Pyrazolylphthalimide derivative **4** was synthesized and reacted with different reagents to afford the target compounds imidazopyrazoles **5-7**, pyrazolopyrimidines **9, 12, 14** and pyrazolotriazines **16, 17** containing phthalimide moiety. The prepared compounds were established by different spectral data and elemental analyses. Additionally, all synthesized derivatives were screened for their antibacterial activity against four types of Gram + ve and Gram-ve strains, and for antifungal activity against two fungi micro-organisms by well diffusion method. Moreover, the antiproliferative activity was tested for all compounds against human liver (HepG-2) cell line in comparison with the reference vinblastine. Moreover, drug-likeness and toxicity risk parameters of the newly synthesized compounds were calculated using *in silico* studies. The data from structure-actvity relationship (SAR) analysis suggested that phthalimide derivative bearing 3-aminopyrazolone moiety, **4** illustrated the best antimicrobial and antitumor activities and might be considered as a lead for further optimization. To investigate the mechanism of the antimicrobial and anticancer activities, enzymatic assay and molecular docking studies were carried out on *E. coli* topoisomerase II DNA gyrase B and VEGFR-2 enzymes.

## Introduction

Nowadays, the most serious public health problems in the world are cancer and infectious diseases^1–3^. The evidence of multi-drug resistant microbial pathogens due to extensive use of antibiotics has been appeared and stimulated the search for discovery of new safer, potent, and resistance-free antimicrobial agents^4^ ^,^ ^5^. Moreover, the research for novel, selective and more potent antitumor agents is still a vital challenge for biologists and medicinal chemists^6^ ^,^ ^7^.

Thalidomide is known as a multi-target drug that affects several cellular processes, including peptidase inhibition, (cyclooxygenase) COX inhibition, glucosidase inhibition and androgen receptor antagonism^8^. Research studies on the structure activity relationship (SAR) of the metabolites and analogues of thalidomide have revealed that the phthalimide ring system is an essential pharmacophoric fragment^9^ ^,^ ^10^. Phthalimide (isoindoline-1,3-dione) has usually been employed in the design of potential antitumour^11^, immunomodulatory^12^, antiangiogenic^13^, anti-microbial^14^ and anti-inflammatory^15^ drug candidates. Further, heterocyclic hits are of considerable utility in synthetic medicines or pesticides and biochemical effects. Heterocycles containing pyrazole, imidazo[1,2-*b*]-pyrazole, pyrazolopyrimidine, pyrazolo-triazine scaffolds exhibit versatile biological properties such as anti-inflammatory, antifungal, antioxidant, antitumor and immunosuppressive agents^16–26^. Hence, molecular hybridization strategy via introduction of different pharmacophoric fragments might improve the biological activity of phthalimide derivatives.

**Figure 1.**
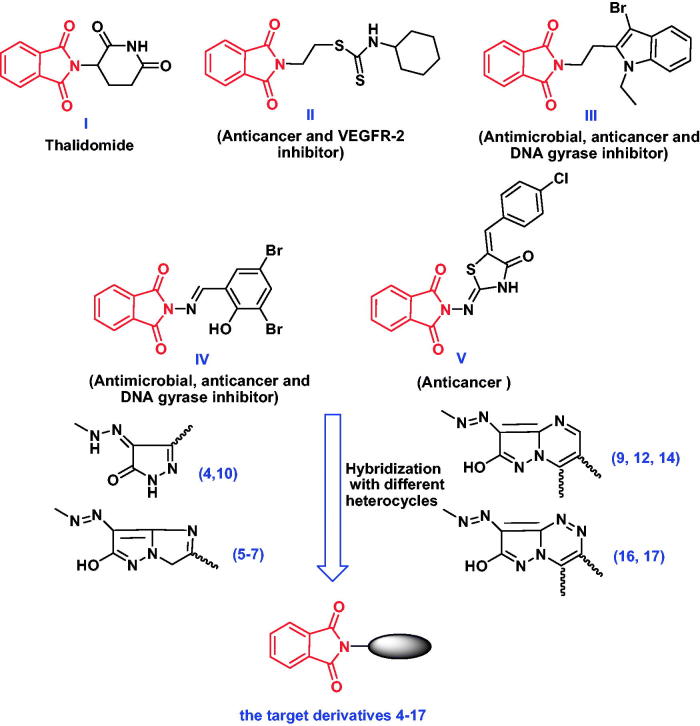
Representative examples of antimicrobial and anticancer agents and structural rationalization of the newly designed compounds **4**–**17**.

Bearing in mind our program in the synthesis of biologically active heterocyclic compounds^27–32^ and the molecular pharmacophores (**I**-**V**) outlined in Figure 1 and their structural requirements^33–36^, some phthalimide derivatives were designed after exploring molecular hybridization approaches with pyrazole, imidazo[1,2-*b*]-pyrazole, pyrazolopyrimidine, pyrazolo-triazine moieties (Figure 1). All the newly prepared phthalimide derivatives were subjected for evaluation of both antimicrobial and anticancer activities with the study of their Drug-Likeness and Toxicity parameters. Furthermore, *in-vitro* enzyme assay of the most potent derivative was performed against *E. coli* topoisomerase II DNA gyrase B and VEGFR-2 enzymes, followed by molecular docking studies to get a distinct insight about the interactions and binding mode in the active sites of these enzymes.

## Experimental

### Chemistry

All melting points were determined on a Gallenkamp apparatus and were uncorrected. The IR spectra were measured on a Pye-UnicamSP300 instrument in potassium bromide discs. The ^1^H-NMR and 13C-NMR spectra were recorded in DMSO-*d_6_* at 400, 500 MHz on JEOL and Broker NMR spectrometer (δ, ppm) using TMS as an internal standard. Mass spectra were obtained on JEOL JMS600 H Root mass spectrometer at 70 ev. Elemental analyses were carried out by the Micro analytical Center of Cairo University, Giza, Egypt. The antimicrobial and anticancer activities were carried out in the Medical Mycology Laboratory of the Regional Center for Mycology and Biotechnology of Al-Azhar University, Cairo, Egypt.

#### Ethyl 2-cyano-2-(2-(1,3-dioxoisoindolin-2-yl)hydrazono)acetate (3)

A solution of 2-aminoisoindoline-1,3-dione (1) (10 mmol) in HCl (3 ml) and water (2 ml) was stirred in ice bath and diazotized with NaNO_2_ (0.3 g, in 5 ml H_2_O). The cold diazonium solution was added to ethyl cyanoacetate (10 mmol) in EtOH (20 ml) containing CH_3_COONa (2 g), was stirred for 2 h. The formed solid was collected by filtration, dried and crystallized from toluene to obtain **3**, (87%) as pale red crystals, m.p. 128–129 °C; IR (KBr) ν cm^−1^ 3355 (NH), 3060 (CH-arom.), 2936 (CH-aliph.), 2225 (CN), 1735, 1713, 1660 (3C=O); ^1^H NMR (DMSO-*d_6_*) δ = 1.22-1.29 (t, *J* = 7.2 Hz, 3H, OCH_2_ CH_3_), 4.17–4.27 (q, *J* = 7.2 Hz, 2H, CH_2_, OCH_2_CH_3_), 7.73–7.80 (m, 4H, Ar-H), 11.12 (s, 1H, NH); 13 C NMR (DMSO-*d_6_)*: 13.5, 60.6, 110.2, 115.3, 124.8, 131.4, 133.3, 162.1, 165.8. Analysis for C_13_H_10_N_4_O_4_ (286.24): Calculated: C, 54.55; H, 3.52; N, 19.57%. Found: C, 54.77; H, 3.76; N, 19.79%.

#### 2-(2-(3-Amino-5-oxo-1H-pyrazol-4(5H)-ylidene)hydrazinyl)isoindoline-1,3-dione (4)

A mixture of compound **3** (2.9 mg, 10 mmol) and NH_2_NH_2_.H_2_O (2 ml) in EtOH (30 ml) was refluxed for 4–6 h, concentrated then cooled. The obtained precipitate was filtered off, dried and crystallized from ethanol to give **4** as brown crystals, yield 83%; m.p. 289–290 °C. IR (KBr): υ cm^−1^ 3422, 3375 and 3268, 3140 (NH_2_ and 2NH), 3080 (CH-arom.), 2945 (CH-aliph.), 1739, 1681, 1661 (3 C=O), 1610 (C=N); ^1^H NMR (DMSO-*d_6_*) δ = 7.75–7.93 (m, 5H, Ar-H + NH), 8.98 (s, 2H, NH_2_), 11.52 (s, 1H, NH)) ; 13 C NMR (DMSO-*d_6_)*: 122.4, 132.6, 135.5, 136.2, 142.8, 163.7, 167.1; MS: *m/z* = 272 [M^+^]. Analysis for C_11_H_8_N_6_O_3_ (272.22): Calculated: C, 48.53; H, 2.96; N, 30.87%. Found: C, 48.74; H, 2.75; N, 30.66%.

### General procedure for the synthesis of imidazo[1,2-b]pyrazole derivatives (5–7)

A mixture of compound **4** (2.7 mg, 10 mmol) and each of ethyl chloroacetate, chloroacetonitrile and phenacyl bromide (10 mmol) was refluxed in DMF (30 ml) containing (0.015 mmol) of NaOH for 5 h. The mixture was poured onto ice and acidified with dilute HCl. The solid obtained were composed by filtration and crystallized from the appropriate solvent to give **5**, **6**, and **7**, respectively.

#### 2-((6-Hydroxy-2-oxo-2,3-dihydro-1H-imidazo[1,2-b]pyrazol-7-yl)diazenyl)-isoindoline-1,3-dione (5)

It was obtained as pale yellow crystals from benzene; yield 73%; m.p. 157–158 °C; IR (KBr): υ cm^−1^ 3500 (OH), 3261 (NH), 3080 (CH-arom.), 2984 (CH-aliph.), 1735, 1685, 1667 (3C=O), 1612 (C=N), 1515 (N=N); ^1^H NMR (DMSO-*d_6_*) δ = 4.62 (s, 2H, CH_2_), 7.70-7.84 (m, 4H, Ar-H), 9.07 (s, 1H, NH), 11.52 (hump, 1H, OH); ^13 ^C NMR (DMSO-*d_6_)*: 65.1, 90.5, 123.2, 132.1, 133.5, 148.0, 160.3, 169.4, 181.6. Analysis for C_13_H_8_N_6_O_4_ (312.24): Calculated: C, 50.01; H, 2.58; N, 26.92%. Found: C, 50.22; H, 2.80; N, 26.71%.

#### 2-((2-Amino-6-hydroxy-3H-imidazo[1,2-b]pyrazol-7-yl)diazenyl)isoindoline-1,3-dione (6)

It was obtained as pale yellow crystals from toluene; yield 69%; m.p. 180–182 °C; IR (KBr): υ cm^−1^ 3500 (OH), 3328, 3144 (NH_2_), 3086 (CH-arom.), 2954 (CH-aliph.), 1740, 1709 (2C=O), 1495 (N=N); ^1^H NMR (DMSO-*d_6_*) δ = 4.02 (s, 2H, CH_2_), 7.72–7.88 (m, 4H, Ar-H), 8.52 (s, 2H, NH_2_), 11.40 (hump, 1H, OH); ^13^C NMR (DMSO-*d_6_)*: 61.3, 104.1, 123.4, 132.0, 133.7, 145.2, 162.1, 167.5, 169.9. Analysis for C_13_H_9_N_7_O_3_ (311.26): Calculated: C, 50.16; H, 2.91; N, 31.50%. Found: C, 50.37; H, 2.69; N, 31.72%.

#### 2-((6-Hydroxy-2-phenyl-1H-imidazo[1,2-b]pyrazol-7-yl)diazenyl)isoindoline-1,3-dione (7)

It was obtained as pale yellow crystals from toluene; yield 76%; m.p. 196–198 °C; IR (KBr): υ cm^−1^ 3504 (OH), 3237 (NH), 3078 (CH-arom.), 2942 (CH-aliph.), 1745, 1710 (2 C=O), 1488 (N=N); ^1^H NMR (DMSO-*d_6_*) δ = 7.35–8.49 (m, 10H, Ar-H + CH-imidazole), 12.02 (s, 1H, NH), 12.75 (s, 1H, OH); ^13^C NMR (DMSO-*d_6_)*: 101.2, 120.0, 123.4, 127.5, 129.7, 130.3, 131.1, 132.4, 133.8, 140.2, 143.0, 160.6, 166.7, 169.5; MS: *m/z* = 372 [M^+^]. Analysis for C_19_H_12_N_6_O_3_ (372.34): Calc. C, 61.29; H, 3.25; N, 22.57%. Found: C, 61.50; H, 3.47; N, 22.78%.

#### 5-Amino-3-((1,3-dioxo-1,3-dihydro-2H-isoindol-2-yl)diazenyl)-2-hydroxy-7-(methylthio)pyrazolo[1,5-a]pyrimidine-6-carbonitrile (9)

A mixture of compound **4** (2.7 mg, 10 mmol) and 2-(bis(methylthio)methylene)malononitrile (1.7 mg, 10 mmol) in DMF (30 ml), in the presence of anhydrous K_2_CO_3_ (1.0 g) refluxed for 7 h. After cooling, it was poured onto ice, and acidified with dilute HCl. The formed solid was filtered off and crystallized from hexane to obtain **9** as buff crystals (55%), m.p 210–211 °C; IR (KBr): υ cm^−1^ 3500 (OH), 3368, 3240 (NH_2_), 3063 (CH-arom.), 2954 (CH-aliph.), 2215 (CN), 1767, 1711 (2 C=O), 1482 (N=N); ^1^H NMR (DMSO-*d_6_*) δ = 2.45 (s, 3H, CH_3_), 7.64–7.87 (m, 6H, Ar-H + NH_2_), 11.80 (s, 1H, OH); ^13^C NMR (DMSO-*d_6_)*: 14.2, 90.1, 101.4, 116.8, 123.3, 131.5, 132.0, 145.2, 153.0, 161.6, 163.4, 169.7. Analysis for C_16_H_10_N_8_O_3_S (394.37): Calculated: C, 48.73; H, 2.56; N, 28.41; S, 8.13%. Found: C, 48.93; H, 2.78; N, 28.63; S, 8.34%.

#### N'-{4-[(1,3-dioxo-1,3-dihydro-2H-isoindol-2-yl)hydrazono]-5-oxo-4,5-dihydro-1H-pyrazol-3-yl}-N,N-dimethylimidoformamide (10)

A solution of **4** (2.7 mg, 10 mmol) in dry dioxane (30 ml) and DMF–DMA (12 mmol) was refluxed for 3 h. After cooling, the solid produced was get by filtration and crystallized from toluene to yield compound **10** as yellow crystals (62%), m.p 136–138 °C; IR (KBr): υ cm^−1^ 3407, 3352 (2NH), 3048 (CH-arom.), 2939 (CH-aliph.), 1733, 1681, 1662 (3 C=O), 1620 (C=N); ^1^H NMR (DMSO-*d_6_*) δ = 2.75 (s, 6H, N(CH_3_)_2_), 7.44–7.82 (m, 5H, Ar-H + NH), 8.01 (s, 1H, N=CH). 12.46 (s, 1H, NH); ^13^C NMR (DMSO-*d_6_)*: 35.8, 123.2, 132.1, 133.7, 136.5, 150.9, 154.4, 164.6, 167.0. Analysis for C_14_H_13_N_7_O_3_ (327.30): Calculated: C, 51.38; H, 4.00; N, 29.96%. Found: C, 51.60; H, 4.20; N, 29.74%.

#### 7-Amino-3-[(1,3-dioxo-1,3-dihydro-2H-isoindol-2-yl)diazenyl]-2-hydroxy-pyrazolo[1,5-a]pyrimidine-6-carbonitrile (12)

A combination of **10** (3.3 mg, 10 mmol) and cyanothioacetamide or malononitrile (10 mmol) in EtOH (30 ml) containing TEA (0.5 ml) was refluxed for 4 h. After cooling, the resulted solid was composed by filtration and crystallized from EtOH to yield compound **12** as brown crystals (66%), m.p 302 °C; IR (KBr): υ cm^−1^ 3500 (OH), 3386, 3248 (NH_2_), 3078 (CH-arom.), 2955 (CH-aliph.), 2220 (CN), 1757, 1712 (2 C=O), 1496 (N=N); ^1^H NMR (DMSO-*d_6_*) δ = 7.60–7.86 (m, 4H, Ar-H), 8.11 (s, 1H, C_5_-H), 9.27 (s, 2H, NH_2_), 12.63 (s, 1H, OH); ^13^C NMR (DMSO-*d_6_)*: 93.6, 101.2, 115.1, 123.6, 132.4, 133.0, 147.7, 160.4, 162.7, 168.1, 170.0. Analysis for C_15_H_8_N_8_O_3_ (348.28): Calculated: C, 51.73; H, 2.32; N, 32.17%. Found: C, 51.94; H, 2.55; N, 32.38%.

#### 2-[(7-Amino-6-benzoyl-2-hydroxypyrazolo[1,5-a]pyrimidin-3-yl)diazenyl]-1H-isoindole-1,3(2H)-dione (14)

A mixture of compound **10** (3.3 mg, 10 mmol) and 3-oxo-3-phenylpropanenitrile (1.5 mg, 10 mmol) in glacial acetic acid (30 ml) was heated under reflux for 4 h. The solid production acquire after cooling was filtered off and crystallized from benzene to give compound **14** as brown crystals (57%), m.p 295–297 °C; IR (KBr): υ cm^−1^ 3502 (OH), 3382, 3241 (NH_2_), 3076 (CH-arom.), 2950 (CH-aliph.), 1746, 1708, 1664 (3 C=O), 1500 (N=N); ^1^H NMR (DMSO-*d_6_*) δ = 7.48-7.89 (m, 9H, Ar-H), 8.05 (s, 1H, C_5_-H), 9.14 (s, 2H, NH_2_), 12.71 (s, 1H, OH); ^13^C NMR (DMSO-*d_6_)*: 100.6, 119.1, 123.8, 128.3, 129.5, 132.4, 133.1, 134.2, 134.9, 147.8, 160.7, 162.0, 164.1, 170.7, 191.3; MS: *m/z* = 427 (75%) [M^+^], 105 (100%) B.P. Analysis for C_21_H_13_N_7_O_4_ (427.37): Calculated: C, 59.02; H, 3.07; N, 22.94%. Found: C, 59.23; H, 3.28; N, 22.72%.

### General procedure for the synthesis of pyrazolo[5,1-c][1,2,4]triazine derivatives 16 and 17

To a cold solution (0–5 °C) of malononitrile, ethyl acetoacetate, and 3-iminobutanenitrile (10 mmol) in EtOH (30 ml) containing CH_3_COONa (2 g), a solution of diazonium chloride **15** ((prepared from 10 mmol of **4** and the appropriate quantities of conc. HCl and sodium nitrite) was added. The reaction combination, in each case, was left at room temperature for 2 h with stirring. The solid result, produced in each case, was filtered off and crystallized from the suitable solvent to yield **16** and **17**, respectively.

#### 4-Amino-8-[(1,3-dioxo-1,3-dihydro-2H-isoindol-2-yl)diazenyl]-7-hydroxy-pyrazolo[5,1-c][1,2,4]triazine-3-carbonitrile (16)

It was obtained as buff crystals from ethanol; yield 67%; m.p. 320 °C; IR (KBr): υ cm^−1^ 3500 (OH), 3327, 3208 (NH_2_), 3062 (CH-arom.), 2938 (CH-aliph.), 2216 (CN), 1747, 1713 (2 C=O), 1497 (N=N); ^1^H NMR (DMSO-*d_6_*) δ = 7.50–8.08 (m, 6H, Ar-H + NH_2_), 13.05 (s, 1H, OH); ^13^C NMR (DMSO-*d_6_)*: 101.7, 115.0, 123.8, 132.2, 133.5, 142.2, 148.4, 150.8, 161.6, 169.1; MS: *m/z* = 350 [M^+^+1]. Analysis for C_14_H_7_N_9_O_3_ (349.26): Calculated: C, 48.14; H, 2.02; N, 36.09%. Found: C, 48.35; H, 2.25; N, 36.30%.

#### Ethyl 8-[(1,3-dioxo-1,3-dihydro-2H-isoindol-2-yl)diazenyl]-7-hydroxy-4-methyl-pyrazolo[5,1-c][1,2,4]triazine-3-carboxylate (17)

It was obtained as pale buff crystals from dioxane; yield 60%; m.p. >300 °C; IR (KBr): υ cm^−1^ 3500 (OH), 3070 (CH-arom.), 2952 (CH-aliph.), 1748, 1718, 1707 (3 C=O), 1492 (N=N); ^1^H NMR (DMSO-*d_6_*) δ = 1.21–1.28 (t, *J* = 7.2 Hz, 3H, OCH_2_ CH_3_), 2.25 (s, 3H, CH_3_), 4.16–4.26 (q, *J* = 7.2 Hz, 2H, CH_2_, OCH_2_CH_3_), 7.62–7.83 (m, 4H, Ar-H), 13.15 (s, 1H, OH); ^13^C NMR (DMSO-*d_6_)*: 12.7, 14.1, 60.9, 100.2, 123.4, 132.3, 133.1, 148.3, 151.8, 155.2, 161.0, 169.6, 170.8. Analysis for C_17_H_13_N_7_O_5_ (395.33): Calculated: C, 51.65; H, 3.31; N, 24.80%. Found: C, 51.86; H, 3.53; N, 24.58%.

### Biological activity

#### Antimicrobial activity

All bacterial and fungal strains were received from the culture collection of the Regional Center for Mycology and Biotechnology (RCMB), Al-Azhar University, Cairo, Egypt. All target derivatives were screened *in-vitro* opposite to various kinds of bacteria, Gram-positive bacteria (*Streptococcus pneumoniae* and *Bacillus subtilis*) and Gram-negative bacteria (*Pseudomonas aeruginosa* and *Escherichia coli*) and for their Antifungal activities against *Aspergillus fumigatus* and *Candida albicans*, respectively. Ampicillin and gentamycin were used as standard antibacterial drugs while amphotericin B was used as reference antifungal drug. The diameter of inhibition zone (mm) was measured for the biological activity using the diffusion technique^37^. The promising compounds were further investigated to evaluate their antimicrobial activity expressed in terms of minimum inhibitory concentration (MIC) using the modified agar well diffusion method^37^.

#### Antitumor activity

The anticancer activity of all derivatives were determined against a human liver cancer cell line (HepG-2) using the 3-(4,5-dimethyl- thiazole-2-yl)-2,5-diphenyl teterazolium bromide (MTT) assay and vinblastine was used as a standard drug following the previously reported procedure^38^ ^,^ ^39^. All experiments were carried out in triplicate.

#### *In-vitro* enzyme assay on DNA gyrase B and VEGFR-2

The *in-vitro* enzyme inhibition assessment for compound **4** (which exhibited the highest potency as antimicrobial and anticancer agent in comparison with the other analogs and the reference drugs) was carried out in confirmatory diagnostic unit, Vacsera, Egypt. The evaluation performed profiling of the compound **4** against *E. coli* DNA gyrase and VEGFR-2 kinases using Novobiocin and Staurosporine as reference drugs, respectively according to the previously reported methods^40^ ^,^ ^41^.

### *In silico* calculations of molecular properties

Molecular descriptors display the pharmacokinetic, pharmacodynamic and physicochemical effects of all synthesized targets **3**–**17**. The lipophilicity (milogP) and topological polar surface area (tPSA) were calculated using the online software Molinspiration^42^, while the aqueous solubility, drug-likeness, drug score were calculated using the OSIRIS property explorer software^43^. Furthermore, according to Veber et al., good bioavailability^44^, is more favorable for targets having TPSA of ≤140 A°2 and ≤10 rotatable bonds. Decreased molecular flexibility, as determined by the rotatable bond number, and low polar surface area or total hydrogen bond count, which are vital predictors of good oral bioavailability, independent of molecular weight.

### Molecular modeling study

Docking study was performed by downloading the Protein Data Bank (PDB) file: 1KZN for *E. coli* topoisomerase II DNA gyrase B^45^ and 2OH4 for VEGFR-2^46^. Verification process was performed by redocking of the co-crystallized ligands into the active sites using Molecular Operating Environment software 10.2008 (MOE)^47^. Then, compound **4** was docked by MOE after preparation of the selected compound through its 3 D protonation and selecting the least energetic conformer using the same reported docking procedure^46^.

## Results and discussion

### Chemistry

**Scheme 1.**
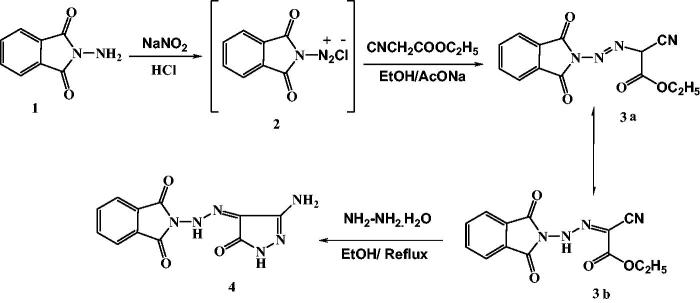
Synthesis of amino pyrazole derivative.

**Scheme 2.**
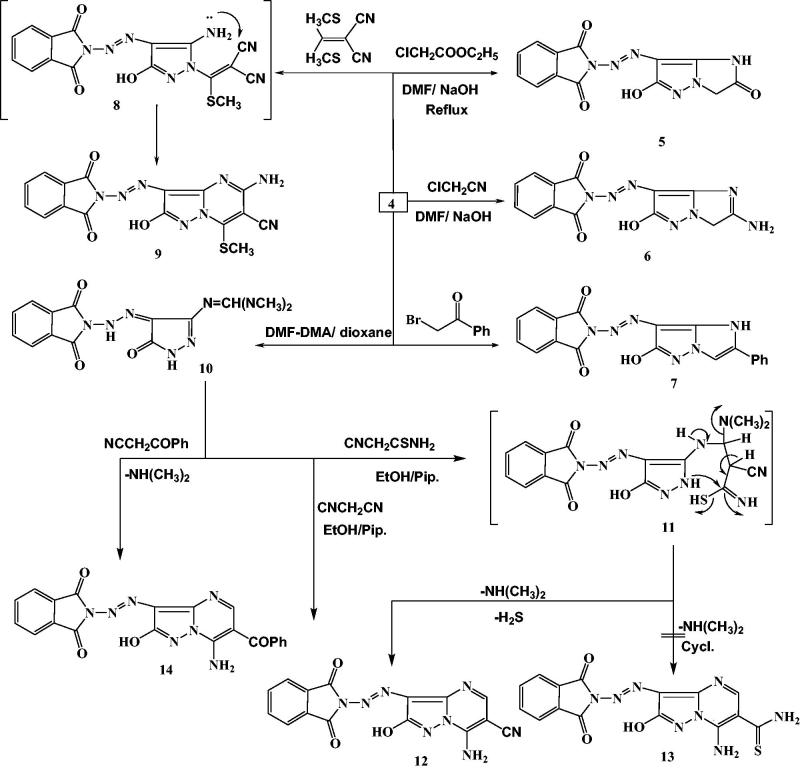
Synthesis of imidazopyrazole and pyrazolopyrimidine derivatives.

**Scheme 3.**
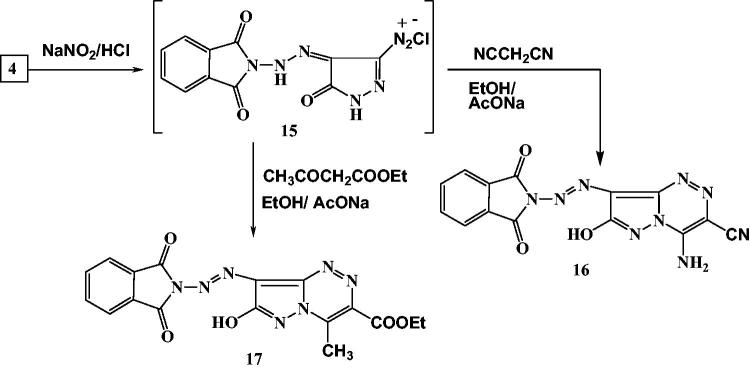
Synthesis of pyrazolotriazine derivatives.

The target derivatives which obtained are showed in Schemes 1–3 based on the synthetic strategies. Synthesis of the precursor hydrazone **3** was achieved by diazotization of *N*-aminophthalimide (**1**)^48^, followed by coupling with ethyl cyanoacetate at room temperature in sodium acetate (Scheme 1). The spectral data confirmed that this compound exists in the hydrazine^49^ form **3 b**, as the ^1^H-NMR and ^13^C NMR spectra (Scheme 1).

2–(2-(3-Amino-5-oxo-1*H*-pyrazol-4(5*H*)-ylidene)hydrazinyl)-isoindoline-1,3-dione (**4**) was synthesized via cyclization of **3** with NH_2_NH_2_.H_2_O under reflux in ethanol. The reaction of **4** with ethyl chloroacetate or chloroacetonitrile in NaOH/DMF solution under reflux yielded the corresponding compounds **5** and **6**, respectively. Treatment of **4** with phenacylbromide afforded imidazopyrazole derivative **7**. 2-(Bis(methylthio)methylene)malononitrile was reacted with compound **4** in the presence of K_2_CO_3_ as a catalyst in DMF under reflux to afford compound **9**. Condensation of **4** with DMF-DMA in dry dioxane under reflux afforded *N*,*N*-dimethylimido-formamide derivative **10**, which was treated with cyanothioacetamide in ethanol/piperidine to afford compound **12**. Moreover, the reaction of **10** with benzoylacetonitrile in refluxing glacial acetic acid yielded product **14** (Scheme 2).

Moreover, aminopyrazole is used for formation of pyrazolotriazine derivatives through diazotization and coupling with active methylene compounds^50^. Aminopyrazole **4** can be diazotized with NaNO_2_ and HCl to give the diazonium salt (intermediate) **15,** which coupled with malononitrile and ethyl acetoacetate in ethanol to yield the pyrazolo[5,1-*c*][1,2,4]triazine derivatives **16** and **17**, respectively (Scheme 3).

### Biological activity

#### Antimicrobial activity

The newly prepared targets were subjected for *in-vitro* antibacterial screening against Gram-positive bacteria (*Streptococcus pneumonia* and *Bacillus subtilis)* and Gram-negative bacteria *(Pseudomonas aeruginosa* and *Escherichia coli)*. Also, these compounds were tested for their antifungal activity against *Aspergillus fumigatus*, and *Candida albicans*. The compounds’ solutions of concentrations (1 mg/mL) were evaluated against the different microorganism’s and the inhibition zone (IZ) used diameter in mm for the biologically activity (agar well diffusion method). The results are depicted in Table 1. From the screening results, we noted that compounds **3, 4, 6, 7, 9, 10, 12, 16** and **17** exhibited significant activity ranging from moderate to excellent against all tested strains except *C. albicans*, showed no activity. Compound **4** showed relatively equipotent inhibition zone as the reference drugs used for different strains, followed by compounds, **9**, **16, 12**, **17**, **6**, **7**, **3** and **10** respectively. The pyrazolyl-*N,N*-dimethylformimidamide derivative **10** exhibited a weak biologically active on both of *B. subtilis* and *A. fumigatus*, while compounds **5** and **14** had no antimicrobial activities.

**Table 1.** Diameter of inhibition zone (mm) of the synthesized compounds at 1 mg/mL.

	Mean diameter of inhibition zone (Mean ± SEM) (mm)					
Compd.	Gram + ve Bacteria		Gram-ve Bacteria		Fungi	
	*S. pneumonia* *RCMB 010010*	*B. subtilis* *RCMB 010067*	*P. aeruginosa* *RCMB 010043*	*E. coli RCMB 010052*	*A. fumigatus* *RCMB 002568*	*C. albicans* *RCMB 005036*
**3**	18.3 ± 0.7	17.3 ± 0.6	15.3 ± 0.7	17.1 ± 0.5	–	–
**4**	23.0 ± 0.4	25.1 ± 0.5	19.2 ± 0.5	24.5 ± 0.3	23.1 ± 0.5	–
**5**	–	–	–	–	–	–
**6**	20.8 ± 1.5	17.8 ± 1.2	11.9 ± 1.3	16.3 ± 0.5	–	–
**7**	12.7 ± 0.6	21.5 ± 1.5	16.9 ± 1.2	11.9 ± 1.3	–	–
**9**	22.2 ± 0.6	23.2 ± 1.5	17.2 ± 1.3	24.0 ± 1.3	16.6 ± 1.3	–
**10**	14.2 ± 0.4	11.3 ± 0.7	12.3 ± 0.4	22.6 ± 1.5	12.1 ± 1.5	–
**12**	18.6 ± 1.2	24.3 ± 0.6	15.8 ± 1.5	21.8 ± 1.3	20.7 ± 1.2	–
**14**	–	–	–	–	–	–
**16**	22.8 ± 0.7	18.8 ± 1.4	18.1 ± 0.6	23.7 ± 0.7	22.9 ± 1.3	–
**17**	21.9 ± 1.2	20.2 ± 1.2	15.5 ± 1.2	20.5 ± 1.3	16.9 ± 1.2	–
**Amphotericin B**	–	–	–	–	23.7 ± 1.1	25.4 ± 0.1
**Ampicillin**	23.8 ± 0.71	26.4 ± 0.50	–	–	–	–
**Gentamycin**	–	–	19.7 ± 0.6	24.9 ± 1.5	–	–

-: No activity under the screening conditions; SEM: standard error mean; each value is the mean of three values.

Furthermore, the most active target structures **4**, **9**, **12**, **16** and **17** were investigated for the assignment of the minimum inhibitory concentration (MIC) (Table 2). Compound **4** explored the best potential MIC values ranged from 0.49 ± 0.39 to 1.95 ± 0.23 µg/mL in comparison with that of the standard compounds, followed by **9**, **16**, **12** and **17** (MIC 3.90 ± 0.01–62.50 ± 0.71 µg/mL).

**Table 2.** Minimum inhibitory concentration (MIC) (μg/mL) of the most active derivatives.

Compd	MIC (Mean ± SEM) (μg/mL)				
	Gram + ve Bacteria		Gram-ve Bacteria		Fungi
	*S. pneumonia* *RCMB 010010*	*B. subtilis* *RCMB 010067*	*P. aeruginosa* *RCMB 010043*	*E. coli* *RCMB 010052*	*A. fumigatus* *RCMB 002568*
**4**	1.95 ± 0.23	0.98 ± 0.42	0.98 ± 0.02	0.49 ± 0.39	1.95 ± 0.28
**9**	3.90 ± 0.20	1.95 ± 1.30	15.63 ± 0.26	0.49 ± 1.08	1.95 ± 0.34
**12**	7.81 ± 1.01	0.98 ± 1.18	62.51 ± 1.06	3.90 ± 0.03	3.90 ± 1.00
**16**	3.90 ± 1.24	7.81 ± 1.16	15.63 ± 1.25	1.95 ± 1.49	3.90 ± 0.39
**17**	3.90 ± 0.01	3.90 ± 1.38	62.50 ± 0.71	3.90 ± 1.24	15.63 ± 1.05
**Amphotericin B**	0.98 ± 0.47	0.49 ± 0.36	–	–	–
**Ampicillin**	–	–	3.90 ± 0.15	0.98 ± 1.05	–
**Gentamycin**	–	–	–	–	1.95 ± 0.03

-: Not tested, SEM: mean of the standard error; each value is the mean of three values.

#### Structure–activity relationship (SAR) for antimicrobial activity

For compound **3** the presence of ethyl 2-cyano-2–(2-hydrazono)acetate moiety at 2-position of isoindoline nucleus improved antibacterial activities against all tested microorganisms (compound **3**). On the other hand, combination of isoindoline nucleus with pyrazole moiety increased the antibacterial activities and antifungal activity against *A. fumigatus* (compound **4**). However, the existence of *N,N*-dimethyl formimidamide substituent decreased the antibacterial and antifungal activities against the tested microorganisms but increased the antibacterial activity against *E. coli* (compound **10**). In the series of substituted imidazo[1,2-*b*]pyrazol-7-yl)diazenyl)-isoindoline **5**–**7**, the presence of amino and phenyl substituents at the position-2 of imidazole ring enhanced the antibacterial activities and showed no activity against the tested fungi (compound **6** and **7**) . In contrast compound **5**, which has oxo group at positon-2, was found to be inactive against all the tested bacteria and fungi. Furthermore, the presence of CN group at position-6 in the pyrazolo[1,5-*a*]-pyrimidine moiety enhanced the antibacterial and antifungal activities (compounds **9** and **12**), while insertion of benzoyl group at position-6 deactivate the tested compound **14**.

#### Anticancer activity

**Figure 2.**
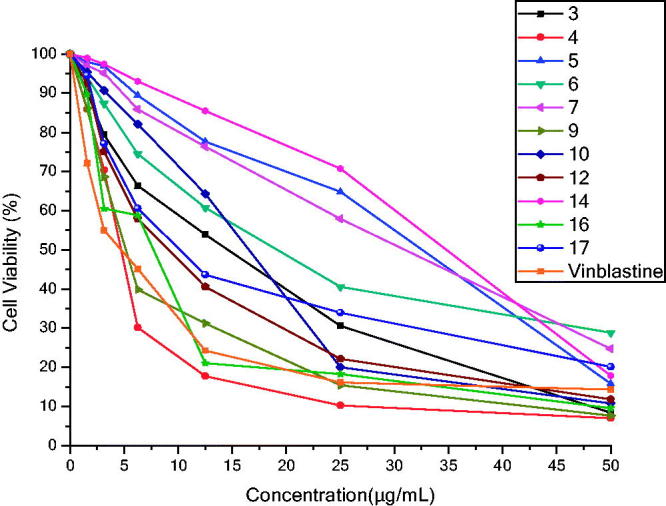
The dose response curve illustrating the inhibitory activity of the tested derivatives **3**, **4**, **5**, **6, 7**, **9**, **10**, **12**, **14**, **16** and **17** against a human liver (HepG-2) cell line compared with the reference drug vinblastine.

All synthesized compounds were screened for their anticancer activity against a human liver (HepG-2) cell line using the 3-(4,5-dimethylthiazole-2-yl)-2,5-diphenyl teterazolium bromide (MTT) assay and vinblastine was used as a standard drug. Cytotoxic activity was depicted in Table 3. Usage of the data to draw a dose-response curve in which the concentrations of the evaluated compounds required to kill fifty percent of cell population (IC_50_) was decided. The results are represented in Table 3 and Figure 2 showed that compound **4** is the most potent cytotoxic derivative and at the same time is relatively equipotent in activity with the reference drug Vinblastine (IC_50_ = 4.22 ± 1.04, 4.63 ± 1.07 µg/mL, respectively). Promising activity was displayed with the following compounds **9 **>** 16 **>** 12 **>** 17**, in a descending order (IC_50_ range 5.60 ± 1.57–9.92 ± 1.28 µg/mL). Also, compounds **3**, **6** and **10** exhibited moderate anticancer activity opposite to the liver carcinoma cell line (HepG-2) (IC_50_ range 12.60 ± 0.13–16.72 ± 0.26 µg/mL). Moreover, compounds **14**, **5**, and **7** were less active among their analogues.

**Table 3.** IC_50_ of the synthesized compounds against cancer HepG-2 and normal THLE-2 liver cell lines.

Comp. No	IC_50_ (Mean ± SEM) (μg/mL)^a^	
	HePG-2	THLE-2
**3**	12.60 ± 0.13	716.37 ± 0.10
**4**	4.22 ± 1.04	874.31 ± 0.22
**5**	31.81 ± 1.56	597.83 ± 0.14
**6**	14.42 ± 0.37	652.46 ± 0.03
**7**	23.61 ± 1.32	662.58 ± 0.17
**9**	5.60 ± 1.57	754.31 ± 0.20
**10**	16.72 ± 0.26	688.29 ± 0.11
**12**	9.11 ± 1.02	793.56 ± 0.31
**14**	34.80 ± 1.26	725.44 ± 0.15
**16**	7.50 ± 0.84	820.58 ± 0.18
**17**	9.92 ± 1.28	658.13 ± 0.25
**Vinblastine**^b^	4.63 ± 1.07	2146.05 ± 0.10

^a^IC_50_: compound concentration which inhibit cell proliferation by 50%.

^b^positive control, SEM: mean of the standard error; each value is the mean of three values.

All compounds were subjected for cytotoxic screening against (THLE-2) normal liver cell line and results demonstrated IC_50_ values (µg/mL) of the synthesized derivatives ranging from 597.83 ± 0.14 to 874.31 ± 0.22, in comparison with the reference (IC_50_ = 2146.05 ± 0.10 µg/mL), respectively. These findings exhibited that all compounds had higher IC_50_ values against normal THLE-2 cells comparing with their IC_50_ doses against the cancer cells (Table 3).

#### Structure–activity relationship (SAR) for cytotoxic activity

It was noticed that there is a great similarity between SAR for antimicrobial activity and that for cytotoxic activity. Substitution at p-2 of isoindoline scaffold with the open chain ethyl 2-cyano-2-(2-hydrazono)acetate moiety in compound **3,** exhibited promising anticancer activity against HepG-2 cell line. Cyclization at p-2 of isoindoline nucleus with 3-aminopyrazolone moiety via hydrazinyl linker in compound **4**, afforded the highest potency among other derivatives. Substituent variation of NH_2_ group at p-3 of pyrazole ring with *N,N*-dimethylimidoformamide group led to remarked decrease in the anticancer activity (compound **10**). Fusion of pyrazole moiety with the five membered ring, imidazole exhibited fall in the cytotoxic activity illustrated in imidazo[1,2-*b*]pyrazole derivatives (**5–7**). However, fusion of pyrazole moiety with the six membered ring, pyrimidine showed elevated activity in 2-hydroxypyrazolo[1,5-*a*]pyrimidine-6-carbonitrile derivatives (**9** and **12**) and drop in the activity in compound **14** substituted with benzoyl moiety at position-6 instead of cyano group. Furthermore, fusion with triazine moiety forming 7-hydroxypyrazolo[5,1-*c*]-[1,2,4]triazine derivatives (**16** and **17**) revealed moderate cytotoxic activity if compared with the reference drug.

#### *In-vitro* enzyme assay on DNA gyrase B and VEGFR-2

The antimicrobial and the cytotoxic results revealed that analog **4** exhibited the highest activity among other analogs. So, subsequent mechanistic studies were supplied through investigating the binding affinity of representative active derivative **4** to *E. coli* DNA gyrase B and VEGFR-2 kinases assaying their effects using suitable positive controls, Novobiocin and Staurosporine, respectively. From Table 4, it was observed that compound **4** represented a nearly equipotent IC_50_ value with Novobiocin as DNA gyrase B inhibitor (IC_50_=0.34 ± 0.63 and 0.28 ± 1.45 µM, respectively). On the other hand, it exhibited excellent and two folds the inhibitory activity of Staurosporine towards VEGFR-2 (IC_50_=0.09 ± 1.30 and 0.17 ± 1.02 µM, respectively).

**Table 4.** Inhibitory evaluation of compound **4** against DNA gyrase B and VEGFR-2 kinases.

Comp. No	IC_50_ (Mean ± SEM) (μM)	
	DNA gyrase B	VEGFR-2
**4**	0.34 ± 0.63	0.09 ± 1.30
**Novobiocin**	0.28 ± 1.45	–
**Staurosporine**	–	0.17 ± 1.02

### *In silico* calculations of molecular properties

#### Drug-Likeness parameters:

Molecular descriptors illustrate the pharmacokinetic, pharmacodynamic and physicochemical properties of the compounds **3**–**17** exhibiting good oral bioavailability of these derivatives theoretically. The calculation results shown in Table 4 revealed that most of the compounds follow the Lipinski rules of the five^51^ ^,^ ^52^, revealing that there would not be problems with oral bioavailability of these compounds theoretically. Expected poor intestinal absorption was accompanied with molecules having TPSA values around 140Å2 or more. Thus, all compounds (except **9**, **12**, **14**, **16** and **17**) have represented a TPSA less than 140Å2, exhibiting a good permeability of the drug in the cellular plasma membrane. It has been shown that for the compound to have a reasonable probability of being well absorbed, miLogP value must be in the range of −0.4 to +5^44^. On this basis, all the synthesized compounds were found to have miLogP values under the acceptable criteria and they are expected to have good oral absorption (Table 5). Also, compounds **9** and **12** have shown very high percentage of absorption (%ABS), that is a parameter of good bioavailability via oral administration but the rest of compounds have a reasonable probability of absorption. Molecules with more than 10 rotatable bonds may have problems with bioavailability^44^. All the tested compounds have **2**–**5** rotatable bonds and they might not have problems with bioavailability. Furthermore, all nOHNH values (H-bond donors) are in the range of 1–4 indicating their solubility in cellular membranes. All compounds having one or zero violation of Lipinski’s rule are expected not to have problems with bioavailability (Table 5), while those violating more than one may have problems with bioavailability^53^.

**Table 5.** Calculated molecular properties of the synthesized compounds for assessment of the drug likeness

Comp. no Rule	m_i_LogP^a^ <5	% ABS^b^	TPSA^c^	N_atoms_^d^	MW^e^ <500	M.Vol.^f^	n_ON_^g^ <10	n_OHNH_^h^ <5	n_viol_.^i^	n_rotb._^j^ (<10)
**3**	1.22	69.83	113.56	21	286.25	237.81	8	1	0	5
**4**	0.25	62.65	135.24	20	272.22	214.71	9	4	0	2
**5**	0.27	63.82	130.96	23	312.25	241.04	10	2	0	2
**6**	1.02	60.62	140.25	23	311.26	244.20	10	3	0	2
**7**	3.40	68.60	117.13	28	372.34	304.08	9	2	0	3
**9**	2.04	86.91	164.05	28	394.38	306.37	11	3	1	3
**10**	0.73	65.94	124.82	24	327.30	272.59	10	2	0	4
**12**	1.15	86.91	164.05	26	348.28	271.68	11	3	1	2
**14**	2.85	54.73	157.32	32	427.38	345.21	11	3	1	4
**16**	1.10	47.96	176.94	26	349.27	267.52	12	3	1	2
**17**	2.16	56.07	153.43	29	395.33	317.26	12	1	1	5
**Amphoter-icin B**	−2.49	1.27	319.61	65	924.09	865.48	18	13	3	3
**Ampicillin**	−0.87	70.11	112.73	24	349.41	298.87	7	4	0	4
**Gentam-icin**	−4.21	40.18	199.74	33	477.60	450.66	12	11	2	7
**Vinblast-ine**	5.56	55.84	154.11	59	810.99	744.65	13	3	3	10

^a^Octanol-water partition coefficient, calculated by the methodology developed by Molinspiration.

^b^% ABS percentage of absorption.

^c^TPSA topological polar surface area.

^d^Number of non-hydrogen atoms.

^e^Molecular weight.

^f^molecular volume.

^g^Number of hydrogen-bond acceptors (O and N atoms).

^h^Number of hydrogen-bond donors (OH and NH groups).

^i^Number of ‘‘Rule of five” violations^.^

^j^Number of rotatable bonds.

The toxicity risk assessment (TRA) indicators, including irritant, tumorigenic, mutagenic and reproductive effects are the tools for the risks of toxicity. This assessment proposed that compounds **5**, **6**, **7**, **9**, **12** and **14** did not show any toxicity risk profile. However, compounds **16** and **17** showed the low of mutagenic and high tumorigenicity effects, respectively. Also, compounds **3**, **4** and **10** have shown the high of irritancy and low reproductive effects, respectively (Table 6). The absorption and distribution characteristics of a compound were significantly affected by its aqueous solubility. It is well known that low solubility is accompanied with bad absorption and the general aim is to be away from the poorly soluble compounds. So, there are more than 80% of the drugs on the market having solubility values greater than −4. Table 6 showed those compounds **3**, **4**, **5**, **6** and **10** exhibiting solubility values above −4 and they are suggested to have good aqueous solubility which significantly influences their absorption and distribution characteristics. Drug-likeness with a positive value points that the derivative consists of fragments involved in most applicable drugs. The drug score merge the risk of toxicity, solubility, lipophilicity, drug-likeness and molecular weight into a single numerical value which can be applied to foretell a global value for each derivative as a potential new drug candidate^54^. The data shown in Table 6 indicated that all compounds have displayed negative values of drug likeness in the comparable zone with that of the standard drugs. The drug score calculation of all compounds revealed positive values ranged from 0.18 to 0.45. Even those compounds with negative drug likeness have illustrated positive drug scores (Table 6). Finally, it could be observed that compounds **5** and **6** have potential as new drug candidates, but the rest of the series have drug scores from low to moderate values comparing with the reference drugs used.

**Table 6.** Toxicity risks, solubility, drug-likeness, and drug score of the target derivatives.

Comp. no.	Toxicity risks				Solubility	Drug-likeness	Drug Score
	Mutagen-icity	Tumorigen-icity	Irritancy	Reproductive effect			
**3**	No risk	No risk	high risk	low risk	−2.6	−12.44	0.22
**4**	No risk	No risk	high risk	low risk	−2.26	−2.18	0.25
**5**	No risk	No risk	No risk	No risk	−3.01	−7.17	0.44
**6**	No risk	No risk	No risk	No risk	−2.81	−7.51	0.45
**7**	No risk	No risk	No risk	No risk	−5.25	−8.23	0.31
**9**	No risk	No risk	No risk	No risk	−5.64	−12.59	0.3
**10**	No risk	No risk	high risk	low risk	−2.32	−0.86	0.28
**12**	No risk	No risk	No risk	No risk	−5.03	−12.32	0.35
**14**	No risk	No risk	No risk	No risk	−6.35	−7.14	0.25
**16**	low risk	high risk	No risk	No risk	−4.6	−12.94	0.18
**17**	low risk	high risk	No risk	No risk	−4.03	−11.35	0.18
**Amphote-ricin B**	No risk	No risk	No risk	No risk	−5.08	−0.14	0.27
**Ampicillin**	No risk	No risk	No risk	No risk	−1.57	10.72	0.91
**Gentamy-cin**	No risk	No risk	No risk	No risk	−1.18	4.88	0.77
**Vinblastine**	No risk	No risk	No risk	No risk	−5.08	5.61	0.35

### Molecular modeling study

Based on the kinase assessment observations and the previous literatures illustrated the important correlation between phthalimide analogs and *E. coli* topoisomerase II DNA gyrase B as antibacterial target^34^ ^,^ ^35^ and VEGFR-2 as anticancer core^32^, we decided to investigate the possible interactions and binding modes of compound **4** with the active sites of those enzymes. Docking simulations were performed using the X-ray crystallographic structures for DNA gyrase B (PDB ID: 1KZN)^44^ with the natural inhibitor clorobiocin and for VEGFR-2 (pdb code: 2OH4)^45^ with the original ligand GIG. The cocrystallized ligands clorobiocin and GIG were redocked into the pocket sites of DNA gyrase B and VEGFR-2 and revealed docking score energies −11.4, −13.7 kcal/mol at RMSD value (root mean square deviation) equal 9.3, 8.5, respectively. The energy is minimized for compound **4** in 3 D picture, and then it saved in a molecular data base (MDB) file to be docked into the active sites of the two enzymes. It showed score energies lower than the co-crystallized ligands (−12.3, −15.6 kcal/mol) with DNA gyrase B and VEGFR-2, respectively.

The binding map of compound **4** in the pocket of DNA gyrase B was explained through two stable hydrogen bonding interactions between the two carbonyl groups of phthalamide scaffold and the sidechains of **Asn46** and **Thr165** (distance: 3.12 and 2.66 Å). Furthermore, the essential amino acid **Asp73** located in the motif II of N-terminal loop provided a hydrogen bond with the NH_2_ proton of pyrazolone moiety (distance: 1.64 Å) (Figure 2).

**Figure: 3.**
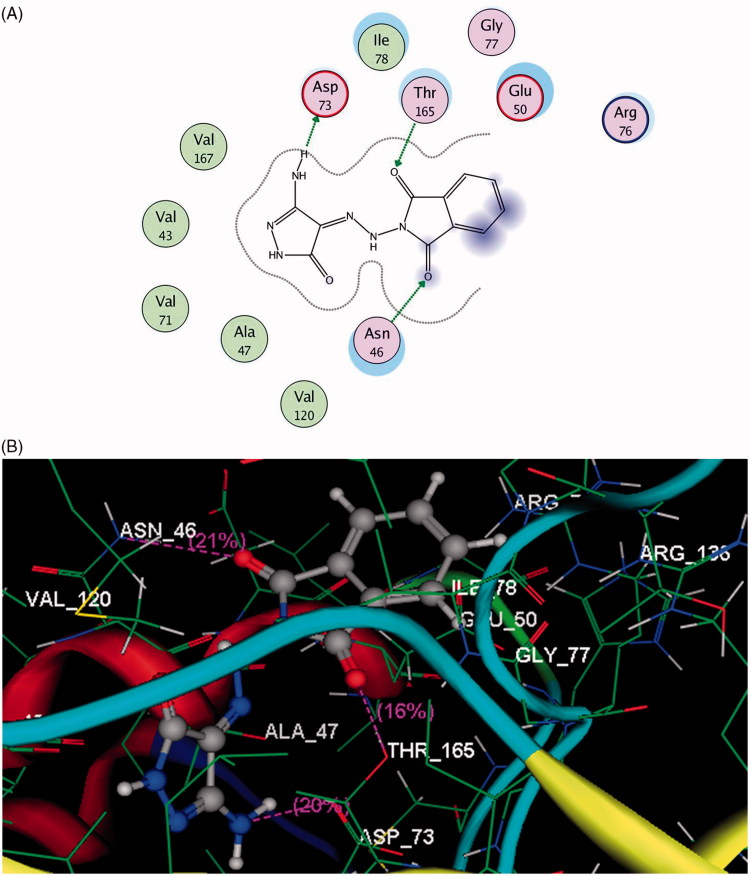
A & B images show 2 D and 3 D docking view of compound **4** in the binding site of DNA gyrase (pdb code: 1KZN), hydrogen bonds are illustrated as dotted purple lines; C atoms are colored gray, N blue and O red.

Docking study results of compound **4** inside the ATP binding site of VEGFR-2 revealed that the carbonyl group of phthalimide moiety formed H-bond acceptor with the backbone of **Asp1044** oriented in the C-terminal domain (distance: 2.18 Å). Furthermore, the two amino protons of pyazolone ring formed H-bond donors with the side chain of Glu883 located in the N-terminal lobe and the backbone of Phe1045 inserted in the C-terminal domain (distance: 1.97, 1.93 Å, respectively) (Figure 3).

**Figure: 4.**
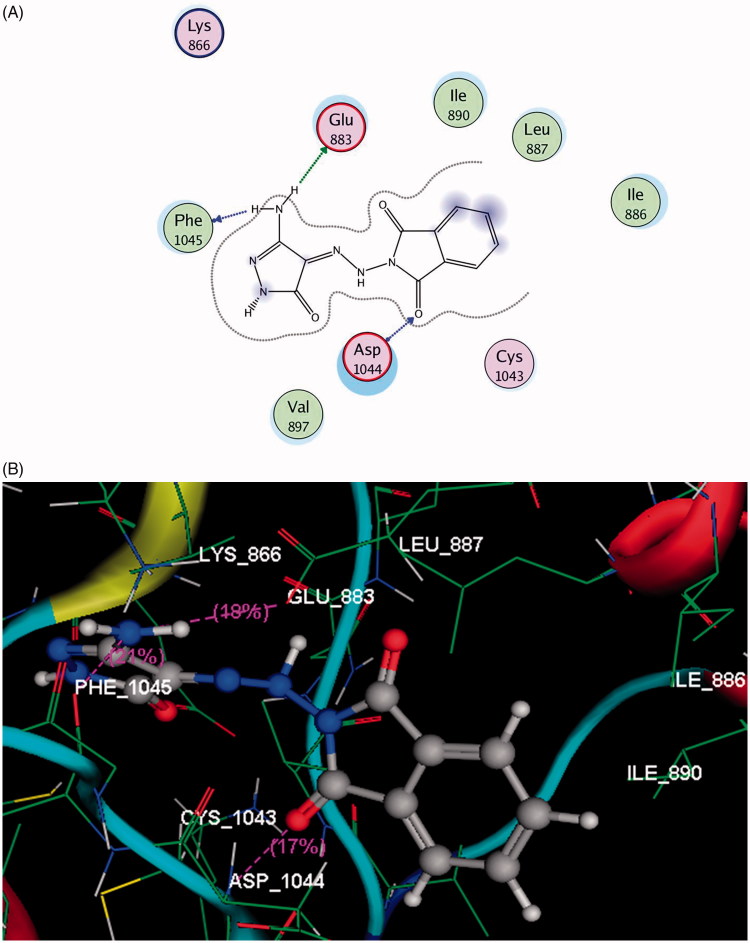
A & B images show 2 D and 3 D docking view of compound **4** in the binding site of VEGFR-2 (pdb code: 2OH4), hydrogen bonds are illustrated as dotted purple lines; C atoms are colored gray, N blue and O red.

Finally, the docking analysis was agreed with the previous antimicrobial, anticancer and enzyme inhibitory activities and could explain how the phthalimide moiety played a pivotal role in stability in the ATP binding sites of both enzymes through its carbonyl groups. Also, the amino group of pyrazolone ring contributed considerably to the strength of binding interactions.

## Conclusion

A new imidazopyrazole, pyrazolopyrimidine and pyrazolo-1,2,4-triazine derivatives containing phthalimide moiety were prepared and *in vitro* antimicrobial and anticancer were reported. Compound **4** was the most active compound against Gram positive bacteria (*S. pneumoniae* and *B. subtilis*), Gram negative bacteria (*P. aeruginosa* and *E. coli*) and fungi (*A. fumigatus*). Also, compound **4** was the most potent compound in cytotoxic assay against hepatic cancer cell line (HepG-2) in comparison with the standard drug vinblastine. Drug-likeness and Toxicity risk parameters of the newly synthesized compounds were calculated using *in silico* studies. The promising results motivated us to perform enzyme assay and docking simulations to gain insight into the plausible mechanism of antibacterial and cytotoxic activities of target compound **4** as DNA gyrase and VEGFR-2 inhibitors. The obtained findings may open up new possibilities for developing a new class of phthalimide drugs with antimicrobial and cytotoxic activity.
